# Hospitalisation patterns of patients with interstitial lung disease in the light of comorbidities and medical treatment – a German claims data analysis

**DOI:** 10.1186/s12931-020-01335-x

**Published:** 2020-03-26

**Authors:** Julia Wälscher, Sabine Witt, Larissa Schwarzkopf, Michael Kreuter

**Affiliations:** 1 Center for Interstitial and Rare Lung Diseases, Pneumology and Respiratory Critical Care Medicine, Thoraxklinik, University of Heidelberg, Member of the German Center for Lung Research (DZL), Röntgenstr. 1, D-69126 Heidelberg, Germany; 2 Institute of Health Economics and Health Care Management, Helmholtz Zentrum München (GmbH) - German Research Center for Environmental Health, Comprehensive Pneumology Center Munich (CPC-M), Member of the German Center for Lung Research (DZL), Heidelberg, Germany

**Keywords:** Interstitial lung disease, Hospitalisation, Comorbidity

## Abstract

**Background:**

Interstitial lung disease (ILD) is a heterogeneous group of mainly chronic lung diseases differing in disease course and prognosis. For most subtypes, evidence on relevance and outcomes of hospitalisations is lacking.

**Methods:**

Using German claims data we investigated number of hospitalisations (zero-inflated-negative-binomial models providing rate ratios (RR)) and time to first hospitalisation (Cox proportional-hazard models providing hazard ratios (RR)) for nine ILD-subtypes. Models were stratified by ILD-related and non-ILD-related hospitalisations. We adjusted for age, gender, ILD-subtype, ILD-relevant comorbidities and ILD-medication (immunosuppressive drugs, steroids, anti-fibrotic drugs).

**Results:**

Among 36,816 ILD-patients (mean age 64.7 years, 56.2% male, mean observation period 9.3 quarters), 71.2% had non-ILD-related and 56.6% ILD-related hospitalisations. We observed more and earlier non-ILD-related hospitalisations in ILD patients other than sarcoidosis. Medical ILD-treatment was associated with increased frequency and in case of late initiation, earlier (non-)ILD-related hospitalisations. Comorbidities were associated with generally increased hospitalisation frequency except for COPD (RR = 0.90) and PH (RR = 0.94) in non-ILD-related and for lung cancer in ILD-related hospitalisations (RR = 0.89). Coronary heart disease was linked with earlier (ILD-related: HR = 1.17, non-ILD-related HR = 1.19), but most other conditions with delayed hospitalisations.

**Conclusion:**

Hospitalisations are frequent across all ILD-subtypes. The hospitalisation risk might be reduced independently of the subtype by improved management of comorbidities and improved pharmacological and non-pharmacological ILD therapy.

## Introduction

Interstitial lung diseases (ILD) represent a heterogeneous, rare group of disorders resulting from damage to the lung parenchyma by varying patterns and causes of inflammation and fibrosis. Owing to their heterogeneous clinical course, the various ILD-subtypes require individualised management strategies [1].

Hospitalisations play a crucial role in disease management – at least for idiopathic pulmonary fibrosis (IPF), the most frequent fibrosing ILD subtype. In IPF, an association between hospitalisations with mortality [2–5], poor clinical outcomes [6] and increased economic burden has been reported. Although some data is available on the impact of hospitalisations in IPF, little is known about the hospitalisation patterns in patients with other ILD subtypes. Moreover, the factors underlying time to and frequency of hospitalisation are poorly understood. This information is paramount to identify patients at risk of hospitalisation and to optimise patient care.

Against this background, this German claims data study provides insight into the pattern and type of hospitalisations for several ILD subtypes. Moreover, it investigates influencing factors for frequency of hospitalisations and time to first hospitalisation for both, ILD-related and non-ILD-related hospitalisations.

## Methods

### Data sample and study population

We performed a retrospective analysis of anonymized patient-level insurance claims data from 2009 to 2014 provided by the Scientific Institute of AOK Statutory Health Insurance funds. The AOK health insurance is one of the largest in Germany insuring about 1/3 of the German population which is judged representative of the German population [7]. Such analyses do not require consultation of an ethics committee [8, 9].

Data included in- and out-patient diagnoses based on ICD-10-codes and procedures undertaken based on International Classification of Procedures in Medicine, respectively, using item codes from the schedule of fees for the outpatient sector. Information on age and gender was also collected.

Study sample of incident ILD patients was detected with a sophisticated process described elsewhere [10] and considered:

- Idiopathic Interstitial Pneumonia (IIP) [J84.1],
- Other Fibrosing ILDs [J84.0, J84.9. D48.1],
- Sarcoidosis [D86.0-D86.9],
- Drug-Associated ILDs [J70.2-J70.4],
- Pneumoconiosis [J62.0-J62.8, J63.0-J63.8 excl. J63.2],
- Radiation-Associated Pneumonitis [J70.1],
- Eosinophilic Pneumonia [J82],
- Hypersensitivity Pneumonitis (HP) [J67.9] and.
- Connective Tissue-associated ILD (CTD) [J99.1].

Within the German health care system outpatient diagnoses are not reported with their exact date but per quarter of the year. Thus, all analyses used quarters as the unit of time.

### Comorbidity burden

Comorbidity burden was assessed by Charlson Index (CI) [11] and the ILD-relevant conditions gastroesophageal reflux disease (GERD), obstructive sleep apnoea syndrome (OSAS), coronary heart disease (CHD), lung cancer, depression, diabetes, chronic obstructive pulmonary disease (COPD), diabetes and pulmonary hypertension (PH) [12–15]. These comorbidities were excluded from CI-categories due to overlap. Comorbidity burden was evaluated by summing the conditions of CI combined with a dummy-coded assessment of the ILD-relevant conditions. Comorbidities (ILD-relevant and sum of CI categories) were defined as present at baseline, if they were documented in the quarter of diagnosis (QDiag). Late onset comorbidity was assumed if a condition was first documented within a follow-up quarter.

### Hospitalisations

Total number of hospitalisations stratified in ILD-related and non-ILD-related hospitalisations was collected quarterly. The following discharge codes were considered ILD-related:

1. ILD (same codes as used for defining the study sample)
2. respiratory infection (A481, B250, J09-J22, J40),
3. pneumothorax (J93),
4. pulmonary embolism (I26),
5. pulmonary hypertension and right heart disease (I50, I270, I272, I278, I279),
6. respiratory insufficiency (J96),
7. other chronic and acute lung diseases (J40-J47).

Hospitalisations with other discharge diagnoses were classified as non-ILD-related.

### Pharmacological and other forms of ILD treatment

Anatomical Therapeutic Chemical (ATC) codes were used to detect the following ILD-related medication: a) systemic steroids, b) immunosuppressants, and c) antifibrotic agents (nintedanib, pirfenidone). Onset of pharmaceutical treatment was classified as “immediate” in case of first prescription within QDiag or the following quarter, “late” in case of first prescription after the first follow up quarter but before the first hospitalisation, and “never” in case of no treatment initiation before the first hospitalisation. Treatment discontinuation was disregarded.

Lung-transplant, invasive ventilation and palliative care during hospitalisations were also assessed.

## Statistical analysis

We assessed baseline characteristics, medication profiles, comorbidity burden, inpatient palliative care, inpatient ventilation, and hospitalisations for the total population and stratified by subtype (Table 1).

**Table 1:** Characterists of hospitalisations at quarter of diagnosis versus follow up period

	Qdiag					>Qdiag				
		ILD-related		non-ILD related			ILD-related		non-ILD-related	
	n	n/mean	%/SD	n/mean	%/SD	n	n/mean	%/SD	n/mean	% / SD
Number of observed individuals	36.816					34.527				
Number of hospitalisations including palliativ care		36	0,2%	118	0,6%		122	0,5%	572	0,6%
Number of hospitalisations with invasive ventilation		1.218	6,6%	1.177	6,3%		2.063	8,6%	2.143	2,3%
In-hospital mortality		883	4,8%	936	5,0%		2.291	9,5%	3.303	3,6%

stratified for ILD and non-ILD related reasons

*SD* standard deviation, *ILD* interstitial lung disease

Number of hospitalisations was analysed with zero-inflated negative binomial regression to consider possible over-dispersion and excess zeros [16]. The covariates of subtype (reference: sarcoidosis), sex (reference: male), number of comorbidities at baseline combined with presence of distinct conditions (yes/no), lung transplant (yes/no), ILD-related medication subtypes (yes/no), age (in years), and number of completely observed follow up quarters were included. Based on Akaike information criterion (AIC) we included age, age^2^, time under observation, and time under observation^2^ for all models, except for the excess zero part of the ILD-related hospitalisation which accounted for time under observation only.

Only individuals surviving QDiag were included in time to first hospitalisation analyses, as we assumed that hospitalisation during QDiag often occurred for diagnostic reasons. We applied Cox regression models adjusted for subtype, age (in years), sex, ILD-related medication subtypes (reference: immediate beginning), number of comorbid conditions and presence of distinct comorbidities as time-dependent variables [17]. Kaplan-Meier Curves, stratified by ILD-subtype, were used to visualise differences in time to hospitalisation.

To evaluate the impact of the several variables, we calculated rate ratios (RR) for number of hospitalisations and hazard ratios (HR) for time to first hospitalisation. All analyses were performed at a significance level of 5% using the software packages SAS, version 9.4.

## Results

### Data sample and study population

We analysed 342,406 completed quarters (mean observation time 9 quarters) in a sample of 36,816 individuals (56.2% male, mean age 64.7 years). The most common diagnosis was IIP (39.3%), followed by sarcoidosis (24.7%), and ‘other fibrosing ILDs’ (19.5%). 20,172 (54.8%) patients received systemic steroids, 2750 (7.5%), immunosuppressants and 399 (1.1%) anti-fibrotic drugs (Table 2).

**Table 2:** Baseline characteristics of the study population

Sample size	36,816
Observed quarters	342,406
Mean (SD) follow up in completed quarters	9.3 (5.7)
Male, n (%) gender	20,703 (56.2)
Mean (SD) age at diagnosis in years	64.7 (14.6)
ILD subtype	
IIP	14,453 (39.3)
OFI	7186 (19.5)
SARC	9106 (24.7)
DAI	407 (1.1)
PNE	1575 (4.3)
RAP	464 (1.3)
EPP	1518 (4.1)
HSP	967 (2.6)
CTD	1140 (3.1)
Quarters between suspected ILD and confirmed ILD, mean (SD)	2.1 (4.6)

*DAI* Drug-Associated ILDs, *CTD* Connective Tissue-associated ILD, *EPP* Eosinophilic Pneumonia, *HSP* Hypersensitivity Pneumonitis, *IIP* Idiopathic Interstitial Pneumonia, *ILD* interstitial lung disease, *OFI* Other Fibrosing ILDs, *PNE* Pneumoconiosis, *RAP* Radiation-Associated Pneumonitis, *SARC* Sarcoidosis

**Fig. 1:**
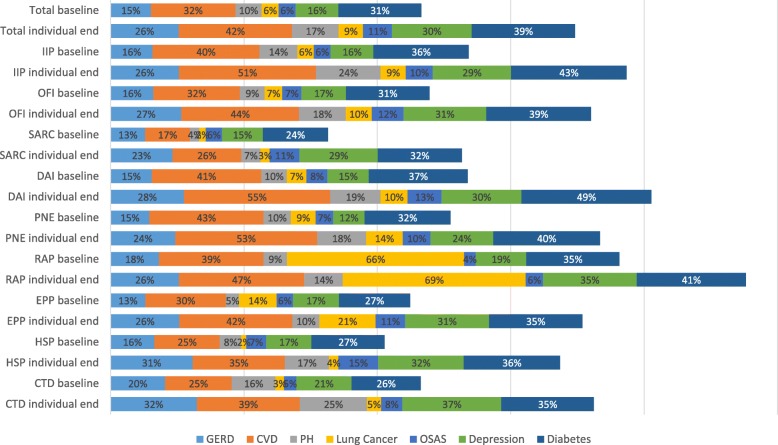
Distribution of comorbidities at baseline vs. individual end of observation period by entity (in percent). IIP Idiopathic Interstitial Pneumonia, OFI Other Fibrosing ILDs, SARC Sarcoidosis, DAI Drug-Associated ILDs, PNE Pneumoconiosis, RAP Radiation-Associated Pneumonitis, EPP Eosinophilic Pneumonia, HSP Hypersensitivity Pneumonitis, CTD Connective Tissue-associated ILD, GERD Gastroesophageal Reflux Disease, CVD coronary vascular disease, PH pulmonary hypertension, OSAS Obstructive Sleep Apnea Syndrome

We observed subtype-specific baseline and longitudinal comorbidity profiles (Fig. 1). CHD (31.6%), diabetes (31.0%) and depression (16.1%) were the most prevalent comorbidities. Until end of follow-up, the prevalence depression increased the most (+ 86.2%), whereas diabetes prevalence increased the lowest (+ 24.7%).

### Types of hospitalisation

A minority of patients were never hospitalised during the observation period (14.0%) while a majority (41.8%) were hospitalised for both, ILD-related and non-ILD-related reasons. Non-ILD related hospitalisations were found in 29.4% and ILD-related hospitalisations in 14.8% (Supplement Table 1).

### Longitudinal hospitalisation pattern

37,304 (24.2%) of the 154,109 hospitalisations occurred in QDiag with similar rates of ILD-related and non-ILD related hospitalisations (49.6% vs. 50.4%). During follow up, the portion of non-ILD-related hospitalisations increased to 79.4%. A detailed quarterwise display of hospitalization frequency is found in Table 3. Reasons for ILD-related hospitalisations varied over time and by subtype (appendix Figure 1). In QDiag, main reasons for hospitalisation was related to ILD itself, followed by respiratory infection (0.3 vs. 0.08 hospitalisations per observed quarter). During follow up, the frequency of hospitalisation resulting from ILD itself and ILD-related respiratory infection were comparable (0.02 vs 0.01).

**Table 3:** Results of the zero-inflated negative binomial regression of frequency of hospitalisations stratified in ILD-related and non-ILD-related

		Non-ILD related		ILD-related	
		Rate ratio	*p*-value	Rate ratio	*p*-value
**Negative binomial model**					
Sex	Male	Reference		Reference	
	Female	**0.96**	0.0009	**0.91**	<.0001
Entity	SARC	Reference		Reference	
	IIP	**1.23**	<.0001	**1.31**	<.0001
	OFI	**1.36**	<.0001	**1.29**	<.0001
	DAI	**1.43**	<.0001	**1.26**	0.0002
	PNE	**1.25**	<.0001	1.06	0.0945
	RAP	**1.63**	<.0001	**1.20**	0.0036
	EPP	**1.43**	<.0001	**1.13**	0.0007
	HSP	1.08	0.0535	1.07	0.1265
	CTD	**2.17**	<.0001	**0.63**	<.0001
OSAS	If present in quarter of diagnosis	**1.11**	<.0001	**1.09**	0.0007
Depression	If present in quarter of diagnosis	**1.09**	<.0001	**1.04**	0.0248
Diabetes	If present in quarter of diagnosis	**1.07**	<.0001	**1.16**	<.0001
GERD	If present in quarter of diagnosis	**1.10**	<.0001	1.00	0.7957
COPD	If present in quarter of diagnosis	**0.90**	<.0001	**1.27**	<.0001
CHD	If present in quarter of diagnosis	**1.15**	<.0001	**1.19**	<.0001
Lung cancer	If present in quarter of diagnosis	**2.04**	<.0001	**0.89**	<.0001
Pulmonary hypertension	If present in quarter of diagnosis	**0.94**	0.001	**1.68**	<.0001
Number of comorbidities	If present in quarter of diagnosis	**1.23**	<.0001	**1.07**	<.0001
Lung transplantation	If occured during observation period	**1.83**	<.0001	**2.36**	<.0001
Immunosuppressive drugs	If treated during observation period	**1.24**	<.0001	**1.24**	<.0001
Anti-fibrotic drugs	If treated during observation period	**0.77**	<.0001	**2.12**	<.0001
Steroids	If treated during observation period	**1.28**	<.0001	**1.95**	<.0001
Age (in years)		**1.02**	<.0001	1.00	0.2924
Age square (in year)		**1.00**	<.0001	**1.00**	0.0055
Time under observation (in quarters)		**1.17**	<.0001	**1.09**	<.0001
Time under observation squared (in quarters)		**1.00**	<.0001	**1.00**	<.0001
**Logistic model**					
Age (in years)		**1.06**	<.0001		
Age square (in year)		**1.00**	<.0001		
Time under observation (in quarters)		**1.07**	0.0005	**1.28**	0.0404
Time under observation squared (in quarters)		**1.00**	0.0008		

Significant estimates are printed in bold

*DAI* Drug-Associated ILDs, *CHD* coronary heart disease, *COPD* chronic obstructive pulmonary disease, *CTD* Connective Tissue-associated ILD, *EPP* Eosinophilic Pneumonia, *GERD* Gastroesophageal Reflux Disease, *HSP* Hypersensitivity Pneumonitis, *IIP* Idiopathic Interstitial Pneumonia, *ILD* interstitial lung disease, *OFI* Other Fibrosing ILDs, *PNE* Pneumoconiosis, *RAP* Radiation-Associated Pneumonitis, *SARC* Sarcoidosis

Across ILD subtypes, the most common reason for non-ILD-related hospitalisations were lung cancer, atrial fibrillation, and CHD (myocardial infarction, angina pectoris, chronic ischaemic heart disease) with a broad subtype-specific variation (Supplement Table 2).

### Influencing factors on frequency of hospitalisations

For non-ILD-related hospitalisations, frequency was increased in ILDs other than sarcoidosis (RR not significant for hypersensitivity pneumonitis). Presence of comorbid conditions was generally associated with more frequent hospitalisation, while COPD and PH were associated with less frequent hospitalisations. Antifibrotic treatment was associated with fewer hospitalisations while steroids and immunosuppressive drugs were associated with an increased frequency of non-ILD-related hospitalisations.

Compared to sarcoidosis patients, patients with pneumoconiosis and hypersensitivity pneumonitis had a similar ILD-related hospitalisation frequency, whereas CTD-patients were hospitalized less frequently. For all other subtypes we observed increased frequencies. Comorbidity burden was generally associated with more ILD-related hospitalisations. Only for lung cancer significantly less ILD-related hospitalisations were observed. All types of medical ILD-treatment were associated with more frequent hospitalisations.

### Influencing factors on time to first hospitalisation during follow up

Compared with sarcoidosis, non-ILD-related hospitalisations occurred earlier in all other subtypes (not significant for pneumoconiosis) (Tables 4). All comorbid conditions were associated with earlier hospitalisation. Late onset treatment with immunosuppressive drugs and steroids was associated with earlier non-ILD-related hospitalisation, whereas absence of steroid treatment was associated with delayed hospitalisation.

**Table 4:** Results of the Cox regression of time to first hospitalisation stratified in ILD-related and non-ILD-related

		Non-ILD related		ILD-related	
		Hazard ratio	*P*-value	Hazard ratio	*P*-value
Sex	Male	Reference		Reference	
	Female	**0.97**	0.0215	**0.86**	<.0001
Age (in years)		**1.01**	<.0001	**1.02**	<.0001
Entity	SARC	Reference		Reference	
	IIP	**1.18**	<.0001	**1.43**	<.0001
	OFI	**1.25**	<.0001	**1.37**	<.0001
	DAI	**1.47**	<.0001	1.17	0.1034
	PNE	1.07	0.0556	1.07	0.2349
	RAP	**1.57**	<.0001	**1.39**	0.0002
	EPP	**1.28**	<.0001	**1.16**	0.0073
	HSP	**1.15**	0.0011	1.06	0.3537
	CTD	**1.91**	<.0001	1.02	0.7829
OSAS	If present or occuring before event	**0.91**	<.0001	**0.96**	0.0165
Depression	If present or occuring before event	**0.95**	<.0001	**0.97**	0.0259
Diabetes	If present or occuring before event	**0.96**	<.0001	**0.93**	<.0001
GERD	If present or occuring before event	**0.96**	<.0001	1.00	0.9959
COPD	If present or occuring before event	0.98	0.1147	**1.35**	<.0001
CHD	If present or occuring before event	**1.17**	<.0001	**1.19**	<.0001
Lung cancer	If present or occuring before event	**0.79**	<.0001	**0.95**	0.0068
Pulmonary hypertension	If present or occuring before event	**0.97**	0.0042	**0.76**	<.0001
Number of comorbidities		**1.18**	<.0001	**1.11**	<.0001
Immunosuppressive drugs	Immediate onset	Reference		Reference	
	Late onset	**1.27**	0.0002	**1.42**	<.0001
	Never	0.98	0.5684	**0.85**	0.0002
Anti-fibrotic drugs	Immediate onset	Reference		Reference	
	Late onset	0.92	0.4798	**2.27**	<.0001
	Never	1.07	0.226	1.03	0.7111
Steroids	Immediate onset	Reference		Reference	
	Late onset	**1.24**	<.0001	**1.64**	<.0001
	Never	**0.91**	<.0001	**0.71**	<.0001

Significant estimates are printed in bold

*DAI* Drug-Associated ILDs, *CHD* coronary heart disease, *COPD* chronic obstructive pulmonary disease, *CTD* Connective Tissue-associated ILD, *EPP* Eosinophilic Pneumonia, *GERD* Gastroesophageal Reflux Disease, *HSP* Hypersensitivity Pneumonitis, *IIP* Idiopathic Interstitial Pneumonia, *ILD* interstitial lung disease, *OFI* Other Fibrosing ILDs, *PNE* Pneumoconiosis, *RAP* Radiation-Associated Pneumonitis, *SARC* Sarcoidosis

In eosinophilic pneumonia, other fibrosing pneumonias, radiation-associated pneumonias and IIP ILD-related hospitalisations occurred earlier than in sarcoidosis. CHD and COPD were associated with earlier hospitalisation but all other conditions were associated with later hospitalisation, with the exception of GERD for which no association was found. Late onset of any ILD-related medication was associated with earlier hospitalisation, in absence of treatment with steroids and immunosuppressive drugs, hospitalisations occurred later.

Kaplan-Meier Curves of time to first non-ILD and ILD-hospitalisation stratified by subtype can be found in the Appendix.

## Discussion

To our knowledge, this is the first study to provide data on hospitalisations in patients with a wide range of ILDs. Data from this large database of patients treated in Germany show that hospitalisation is remarkably frequent, and associated with a wide range of reasons, which, vary by ILD subtype.

Over the 5 years observation period of our study, the majority of patients (86%) were hospitalised, even though not predominately because of ILD-related reasons. These rates substantially exceed those reported for IPF-patients in the United States (23% [6]) and other reports from Germany (50% [18]), but are in line with data from a study conducted in France (87% [19]). An explanation might be that the different rates observed can be explained by the different patient populations in each study and by different hospitalisation patterns. Previous studies with lower rates of hospitalisation were conducted in patients with idiopathic pulmonary fibrosis. In these patients, inpatient admission is not required for diagnosis, as some radiological patterns do not require histological proof [20]. Conversely, a differential diagnosis of another ILD subtype is often performed based on histological examination stemming from transbronchial biopsy in many countries, including Germany and this requires inpatient admission.

Our hypothesis of the impact of inpatient ILD diagnostics on hospitalisation rates is supported by an equal share of ILD-related and non-ILD-related hospitalizations during the QDiag followed by a declining proportion of ILD-related hospitalisations during follow up. This profile might be explained by good outpatient management of ILDs, and/or potential curative success in some ILD subtypes such as sarcoidosis. Thus the frequency of non-ILD-related hospitalisation increases artificially. Furthermore, death of severely diseased individuals who are per se hospitalized more often than less advanced patients, should be taken into account.

Subtype-specific hospitalisation profiles mirror the course of the underlying ILD. For example, in line with the comparably good prognosis and the low comorbidity burden related to the younger age of patients with sarcoidosis [21], a quarter of these patients were never hospitalised and half of them had no ILD-related hospitalisations. In contrast, radiation-associated and drug-associated ILDs, which reflect sequelae of e.g. tumour-directed therapy, presented the highest percentage of patients with ILD-related plus non-ILD-related hospitalisation. In these patients, non-ILD-related hospitalisation were mainly cancer-related (lung cancer, Morbus Hodkin, secondary malignant neoplasms, follow up examinations after tumour-directed therapy), underpinning that in these cases ILD adds to an already substantial morbidity burden. Finally, just over a third of CTD patients had ILD-related hospitalisations, but almost all had non-ILD-related ones. This might reflect that either the lung-component of CTD is well managed via outpatient drug therapy, whereas the systemic dimension of the disease results in severe complications – in our cases mainly ‘necrotizing vasculopathies’, ‘systemic sclerosis’ and ‘systemic lupus erythematosus’- that require frequent inpatient treatment.

A possible limitation of this work is that the ICD-10 coding does not reflect the actual disease.

Comorbidity burden increased hospitalisation frequency and the likelihood of earlier hospitalisation. This effect was more pronounced in non-ILD-related hospitalisations. Unexpectedly, OSAS, depression, diabetes and, to lesser extent GERD, were associated with more frequent but later hospitalisations. We hypothesise that patients with these comorbidities have a high outpatient treatment pattern not require the need for hospitalisation. Thus, complications that could result in hospitalisation might be detected earlier and/or prevented. However, management of complications might be complicated by the patients’ more severe morbidity profile resulting in more frequent hospitalisations.

Lung cancer was associated with less frequent ILD-related but more frequent non-ILD-related hospitalisations, both of which were delayed. We consider this observation a reimbursement-related artefact. A primary diagnosis of lung cancer generally results in a higher reimbursement than a primary diagnosis of ILD, which potentially biases a coding of lung cancer as the primary diagnosis. For PH and COPD the pattern of hospitalisation was reversed; these conditions were associated with more frequent ILD-related hospitalisations but less frequent non-ILD-related hospitalisations. Regarding PH our decision to classify hospitalisations in context of PH and right-heart disease as ILD-related (to account for ILD-sequelae potential misclassification) is the most plausible explanation. Thus, hospitalisations owing to PH contribute to ILD-related rather than non-ILD-related stays. A similar effect is likely to have occurred for COPD, since acute and chronic respiratory conditions were also classified as ILD-related.

Focussing on medical ILD treatment, anti-fibrotic drugs were associated with fewer non-ILD-related but more ILD-related hospitalisations, as previously described for IPF [9]. However, in our sample only 1% of patients received anti-fibrotic therapy, because pirfenidone was only licensed for use by the German Statutory Health Insurance during our observation period and nintedanib became available even later. It is possible that physicians’ lack of experience with antifibrotic agents at that time might have biased prescription only to patients with a better performance status – which therefore led to a low non-ILD-related hospitalisation rate.

Regarding treatment onset, later initiation was linked to earlier hospitalisations This might mirror insufficient (outpatient) management of ILDs in non-specialised centres resulting in acute respiratory deterioration as medical treatment was potentially initiated too late to prevent hospitalisation. In line with this hypothesis, absence of ILD-related medication – which was associated with delayed hospitalisation – might reflect a subgroup with a mild symptom profile. Unfortunately, due to the nature of this research, we were not able to assess the severity of ILD in our analysis and therefore cannot verify our hypothesis.

The findings of our analyses have to be interpreted against some limitations, inherent to retrospective observational claims data based studies. Firstly, the current ICD-10 coding system is not sufficiently differentiated to precisely distinct fibrosing ILDs; as such, patients in these groups may be substantially heterogeneous. Secondly, claims data do not include information on lifestyle factors such as smoking and physical activity that are known to influence outcomes in patients with respiratory disorders. Thirdly, our analysis of ILD-relevant conditions did not consider potential interactions between comorbidity and subtype, owing to issues of model convergence. Thus, we cannot exclude that some conditions affect some subtypes more detrimentally than others.

Despite these drawbacks, this is the first analysis to investigate hospitalisation and its influencing factors for a broad population of patients with various ILDs. The patients included can be considered representative of the German population [22]. Moreover, we separated ILD-related and non-ILD-related hospitalisations, whilst most previous studies either focussed on ILD-related or all-cause hospitalization [18, 19] which could potentially mask the impact of comorbidity and medical treatment. Furthermore, we included analysis of time to hospitalization and hospitalization frequency, to elucidate potential influences in opposite directions.

## Conclusion

Our analyses suggest that hospitalisations are frequent across all ILD-subtypes. Irrespective of subtype, advanced comorbidity-management and stringent pharmaceutical ILD therapy reduce hospitalisation risk [9]. These findings warrant further prospective confirmation and might be used to better understand, and optimise, the care of patients with ILDs.

## Supplementary information

**Additional file 1: Figure 1.** Discharge diagnosis of ILD-related hospitalisation in quarter of diagnosis or later stratified by ILD-subtype displayed as rates (hospitalisations per observed quarters).

**Additional file 2: Figure 2.** Time (in months) to first non-ILD hospitalisation stratified by entity.

**Additional file 3: Figure 3.** Time (in months) to first ILD-related hospitalisation stratified by entity.

**Additional file 4: Table 1.** Hospitalisations during whole observation period.

**Additional file 5: Table 2.** Main reasons for non-ILD related hospitalisations during whole observation period displayed as rank within each ILD-subtype.

## Data Availability

The datasets used and/or analysed during the current study are available from the corresponding author on reasonable request.

## References

[1] American Thoracic Society/European Respiratory Society International Multidisciplinary Consensus Classification of the Idiopathic Interstitial Pneumonias (2002). This joint statement of the American Thoracic Society (ATS), and the European Respiratory Society (ERS) was adopted by the ATS board of directors, June 2001 and by the ERS Executive Committee, June 2001. Am J Respir Crit Care Med.

[2] Tachikawa R, Tomii K, Ueda H, Nagata K, Nanjo S, Sakurai A (2012). Clinical features and outcome of acute exacerbation of interstitial pneumonia: collagen vascular diseases-related versus idiopathic. Respiration..

[3] Hozumi H, Nakamura Y, Johkoh T, Sumikawa H, Colby TV, Kono M (2013). Acute exacerbation in rheumatoid arthritis-associated interstitial lung disease: a retrospective case control study. BMJ Open.

[4] Hariri LP, Mino-Kenudson M, Shea B, Digumarthy S, Onozato M, Yagi Y (2012). Distinct histopathology of acute onset or abrupt exacerbation of hypersensitivity pneumonitis. Hum Pathol.

[5] Collard HR, Moore BB, Flaherty KR, Brown KK, Kaner RJ, King TE (2007). Acute exacerbations of idiopathic pulmonary fibrosis. Am J Respir Crit Care Med.

[6] Brown AW, Fischer CP, Shlobin OA, Buhr RG, Ahmad S, Weir NA (2015). Outcomes after hospitalization in idiopathic pulmonary fibrosis. Chest..

[7] Jaunzeme J, Eberhard S, Geyer S (2013). How “representative” are SHI (statutory health insurance) data? Demographic and social differences and similarities between an SHI-insured population, the population of Lower Saxony, and that of the Federal Republic of Germany using the example of the AOK in Lower Saxony. Bundesgesundheitsbl Gesundheitsforsch Gesundheitsschutz.

[8] Swart E, Gothe H, Geyer S, Jaunzeme J, Maier B, Grobe TG (2015). Good practice of secondary data analysis (GPS): guidelines and recommendations. Gesundheitswesen..

[9] Ley B, Swigris J, Day BM, Stauffer JL, Raimundo K, Chou W (2017). Pirfenidone reduces respiratory-related hospitalizations in idiopathic pulmonary fibrosis. Am J Respir Crit Care Med.

[10] Schwarzkopf L, Witt S, Waelscher J, Polke M, Kreuter M (2018). Associations between comorbidities, their treatment and survival in patients with interstitial lung diseases - a claims data analysis. Respir Res.

[11] Charlson ME, Pompei P, Ales KL, MacKenzie CR (1987). A new method of classifying prognostic comorbidity in longitudinal studies: development and validation. J Chronic Dis.

[12] Raghu G, Amatto VC, Behr J, Stowasser S (2015). Comorbidities in idiopathic pulmonary fibrosis patients: a systematic literature review. Eur Respir J.

[13] Margaritopoulos GA, Antoniou KM, Wells AU. Comorbidities in interstitial lung diseases. Eur Respir Rev. 2017;26(143):160027. 10.1183/16000617.0027-2016. Print 2017 Jan. 10.1183/16000617.0027-2016 PMC9488735 28049126

[14] Fulton BG, Ryerson CJ (2015). Managing comorbidities in idiopathic pulmonary fibrosis. Int J Gen Med.

[15] Kreuter M, Ehlers-Tenenbaum S, Palmowski K, Bruhwyler J, Oltmanns U, Muley T (2016). Impact of comorbidities on mortality in patients with idiopathic pulmonary fibrosis. PLoS One.

[16] Greene W (1994). Accounting for excess zeros and sample selection in poisson and negative binomial regression models.

[17] Fisher LD, Lin DY (1999). Time-dependent covariates in the cox proportional-hazards regression model. Annu Rev Public Health.

[18] Behr J, Kreuter M, Hoeper MM, Wirtz H, Klotsche J, Koschel D (2015). Management of patients with idiopathic pulmonary fibrosis in clinical practice: the INSIGHTS-IPF registry. Eur Respir J.

[19] Cottin V, Schmidt A, Catella L, Porte F, Fernandez-Montoya C, Le Lay K (2017). Burden of idiopathic pulmonary fibrosis progression: a 5-year longitudinal follow-up study. PLoS One.

[20] Raghu G, Remy-Jardin M, Myers JL, Richeldi L, Ryerson CJ, Lederer DJ (2018). Diagnosis of idiopathic pulmonary fibrosis. An Official ATS/ERS/JRS/ALAT clinical practice guideline. Am J Respir Crit Care Med.

[21] Arkema EV, Cozier YC (2018). Epidemiology of sarcoidosis: current findings and future directions. Ther Adv Chronic Dis.

[22] Jaunzeme J, Eberhard S, Geyer S (2013). Wie “repräsentativ” sind GKV-Daten?. Bundesgesundheitsbl Gesundheitsforsch Gesundheitsschutz.

