# Supplementary material for: Hospitalisation patterns of patients with interstitial lung disease in the light of comorbidities and medical treatment – a German claims data analysis
Source: Respir Res. 2020 Mar 26;21:73. doi: 10.1186/s12931-020-01335-x (PMC7098099; doi:10.1186/s12931-020-01335-x)
Supplement: Supplementary file 1 — Additional file 1: Figure 1. Discharge diagnosis of ILD-related hospitalisation in quarter of diagnosis or later stratified by ILD-subtype displayed as rates (hospitalisations per observed quarters). [file 12931_2020_1335_MOESM1_ESM.docx]

Supplement

| Figure 1. Discharge diagnosis of ILD-related hospitalisation in quarter of diagnosis or later stratified by ILD-subtype displayed as rates (hospitalisations per observed quarters) |
| --- |
|  |
| DAI, Drug-Associated ILDs; CTD, Connective Tissue-associated ILD; EPP, Eosinophilic Pneumonia; HSP, Hypersensitivity Pneumonitis; IIP, Idiopathic Interstitial Pneumonia; ILD, interstitial lung disease; OFI, Other Fibrosing ILDs; PH, pulmonary hypertension; PNE, Pneumoconiosis; RAP, Radiation-Associated Pneumonitis; RH, right heart; SARC Sarcoidosis. |
