# Supplementary figures and images for: Hospitalisation patterns of patients with interstitial lung disease in the light of comorbidities and medical treatment – a German claims data analysis
Source: Respir Res. 2020 Mar 26;21:73. doi: 10.1186/s12931-020-01335-x (PMC7098099; doi:10.1186/s12931-020-01335-x)

Figure 2: Time (in months) to first non-ILD hospitalisation stratified by entity


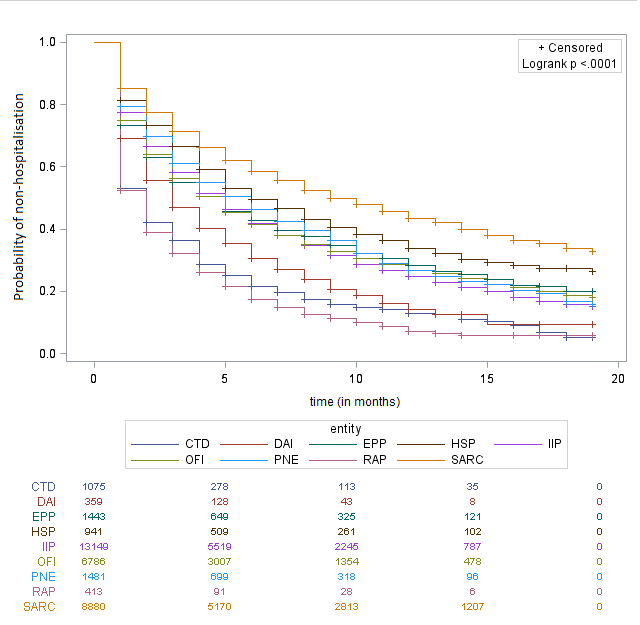

Supplement: Supplementary file 2 — Additional file 2: Figure 2. Time (in months) to first non-ILD hospitalisation stratified by entity. [file 12931_2020_1335_MOESM2_ESM.docx]

Figure 3: Time (in months) to first ILD-related hospitalisation stratified by entity


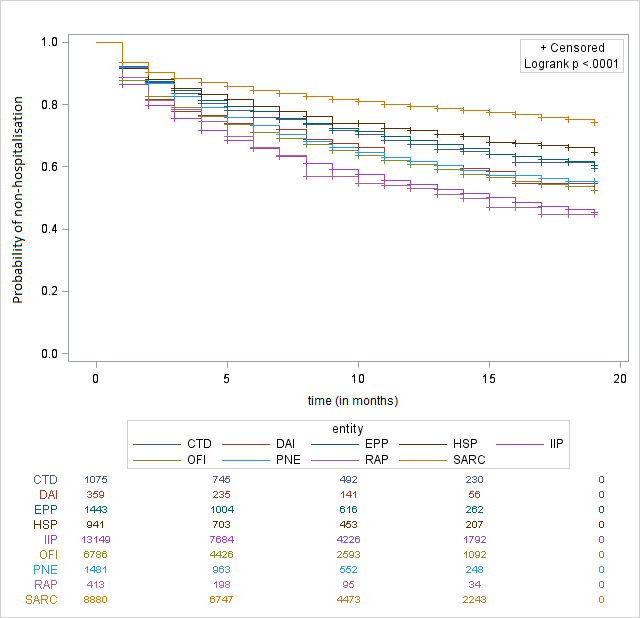

Supplement: Supplementary file 3 — Additional file 3: Figure 3. Time (in months) to first ILD-related hospitalisation stratified by entity. [file 12931_2020_1335_MOESM3_ESM.docx]
