# Supplementary material for: Hospitalisation patterns of patients with interstitial lung disease in the light of comorbidities and medical treatment – a German claims data analysis
Source: Respir Res. 2020 Mar 26;21:73. doi: 10.1186/s12931-020-01335-x (PMC7098099; doi:10.1186/s12931-020-01335-x)
Supplement: Supplementary file 4 — Additional file 4: Table 1. Hospitalisations during whole observation period. [file 12931_2020_1335_MOESM4_ESM.docx]

supplement

Table 1: Hospitalisations during whole observation period

| n (%) | Never hospitalised | Reason for hospitalisation | | |
| --- | --- | --- | --- | --- |
|  |  | Non-ILD | ILD | Both |
| Total (n=36816) | 5156 (14.0) | 10839 (29.4) | 5435 (14.8) | 15386 (41.8) |
| Subtype | | | | |
| IIP (n=14453) | 1493 (10.3) | 3993 (27.6) | 2397 (16.6) | 6570 (45.5) |
| OFI (n=7186) | 744 (10.4) | 1987 (27.7) | 1100 (15.3) | 3355 (46.7) |
| SARC (n=9106) | 2226 (24.5) | 2530 (27.8) | 1395 (15.3) | 2955 (32.5) |
| DAI (n=407) | 30 (7.4) | 102 (25.1) | 55 (13.5) | 220 (54.1) |
| PNE (n=1575) | 218 (13.8) | 545 (34.6) | 170 (10.8) | 642 (40.8) |
| RAP (n=464) | 15 (3.2) | 196 (42.2) | 27 (5.8) | 226 (48.7) |
| EPP (n=1518) | 229 (15.1) | 547 (36.0) | 151 (10.0) | 591 (38.9) |
| HSP (n=967) | 179 (18.5) | 258 (26.7) | 125 (12.9) | 405 (41.9) |
| CTD (n=1140) | 22 (1.9) | 681 (59.7) | 15 (1.3) | 422 (37.0) |

DAI, Drug-Associated ILDs; CTD, Connective Tissue-associated ILD; EPP, Eosinophilic Pneumonia; HSP, Hypersensitivity Pneumonitis; IIP, Idiopathic Interstitial Pneumonia; ILD, interstitial lung disease; OFI, Other Fibrosing ILDs; PNE, Pneumoconiosis; RAP, Radiation-Associated Pneumonitis; SARC, Sarcoidosis.
