# Supplementary material for: Hospitalisation patterns of patients with interstitial lung disease in the light of comorbidities and medical treatment – a German claims data analysis
Source: Respir Res. 2020 Mar 26;21:73. doi: 10.1186/s12931-020-01335-x (PMC7098099; doi:10.1186/s12931-020-01335-x)
Supplement: Supplementary file 5 — Additional file 5: Table 2. Main reasons for non-ILD related hospitalisations during whole observation period displayed as rank within each ILD-subtype. [file 12931_2020_1335_MOESM5_ESM.docx]

supplement

Table 2: Main reasons for non-ILD related hospitalisations during whole observation period displayed as rank within each ILD-subtype

| Discharge entity - non-ILD hospitalisation | ICD-10 | IIP | OFI | SARC | DAI | PNE | RAP | EPP | HSP | CTD |
| --- | --- | --- | --- | --- | --- | --- | --- | --- | --- | --- |
|  |  | rank | rank | rank | rank | rank | rank | rank | rank | rank |
| Malignant neoplasm of bronchus and lung | C34 | 1 | 1 | 1 | 1 | 1 | 1 | 1 | 4 | 7 |
| Atrial fibrillation and flutter | I48 | 2 | 3 | 2 | 2 | 6 | 5 | 3 | 3 | 12 |
| Acute myocardial infarction | I21 | 5 | 9 | 12 | 9 | 2 | 7 | 12 | 6 | 15 |
| Angina pectoris | I20 | 3 | 6 | 6 | 25 | 4 | 13 | 5 | 5 | 18 |
| Chronic ischaemic heart disease | I25 | 4 | 5 | 9 | 11 | 3 | 16 | 7 | 7 | 26 |
| Sleep disorders | G47 | 15 | 13 | 4 | 17 | 21 | 20 | 13 | 1 | 35 |
| Diabetes mellitus, Type 2 | E11 | 7 | 10 | 3 | 42 | 19 | 22 | 11 | 17 | 45 |
| Neoplasm of uncertain or unknown behaviour of middle ear and respiratory and intrathoracic organs | D38 | 10 | 8 | 8 | 41 | 5 | 9 | 2 | 64 | 39 |
| Secondary malignant neoplasm of other and unspecified sites | C79 | 36 | 12 | 70 | 24 | 48 | 2 | 8 | 107 | 97 |
| Failure and rejection of transplanted organs and tissues | T86 | 11 | 14 | 133 | 88 | 423 | - | 47 | 2 | 16 |
| Systemic sclerosis | M34 | 18 | 117 | 190 | 111 | 378 | - | - | - | 2 |
| Hodgkin lymphoma | C81 | 129 | 76 | 23 | 3 | 104 | 144 | 325 | - | 27 |
| Systemic lupus erythematosus | M32 | 238 | 229 | 345 | - | 86 | - | - | - | 3 |
| Other necrotizing vasculopathies | M31 | 98 | 73 | 159 | 35 | 260 | - | 232 | 121 | 1 |
| Follow-up examination after treatment for malignant neoplasms | Z08 | 102 | 181 | 219 | 65 | 78 | 3 | 38 | - | 448 |
| Malignant neoplasm of other connective and soft tissue | C49 | 334 | 2 | 185 | 54 | 191 | - | 101 | 177 | 247 |

DAI, Drug-Associated ILDs; CTD, Connective Tissue-associated ILD; EPP, Eosinophilic Pneumonia; HSP, Hypersensitivity Pneumonitis; IIP, Idiopathic Interstitial Pneumonia; ILD, interstitial lung disease; OFI, Other Fibrosing ILDs; PNE, Pneumoconiosis; RAP, Radiation-Associated Pneumonitis; SARC Sarcoidosis.
